# GeSICA: Genome segmentation from intra-chromosomal associations

**DOI:** 10.1186/1471-2164-13-164

**Published:** 2012-05-04

**Authors:** Lin Liu, Yiqian Zhang, Jianxing Feng, Ning Zheng, Junfeng Yin, Yong Zhang

**Affiliations:** 1 Department of Bioinformatics, School of Life Science and Technology, Tongji University, Shanghai, 200092, China; 2 Department of Mathematics, Tongji University, Shanghai, 200092, China

## Abstract

**Background:**

Various aspects of genome organization have been explored based on data from distinct technologies, including histone modification ChIP-Seq, 3C, and its derivatives. Recently developed Hi-C techniques enable the genome wide mapping of DNA interactomes, thereby providing the opportunity to study genome organization in detail, but these methods also pose challenges in methodology development.

**Results:**

We developed Genome Segmentation from Intra Chromosomal Associations, or GeSICA, to explore genome organization and applied the method to Hi-C data in human GM06990 and K562 cells. GeSICA calculates a simple logged ratio to efficiently segment the human genome into regions with two distinct states that correspond to rich and poor functional element states. Inside the rich regions, Markov Clustering was subsequently applied to segregate the regions into more detailed clusters. The binding sites of the insulator, cohesion, and transcription complexes are enriched in the boundaries between neighboring clusters, indicating that inferred clusters may have fine organizational features.

**Conclusions:**

Our study presents a novel analysis method, known as GeSICA, which gives insight into genome organization based on Hi-C data. GeSICA is open source and freely available at: http://web.tongji.edu.cn/~zhanglab/GeSICA/

## Background

Upon studying the function of the eukaryotic nucleus, genome organization can be used to modulate the interpretation of functional information encoded by the primary DNA sequence [1,2]. It has been suggested that different organizational units reside in distinct chromatin environments and thereby contribute to genomic functional diversity [3-5]. Over the past several years, genome organization has been explored based on data using a variety of technologies. One type of approaches is based on genomic data, such as histone modification (ChIP-Seq) or chromatin components (DamID), to segment the whole genome into elaborate organizational units (called as states or domains) with the computational frameworks such as Hidden Markov Models or Bayesian networks [6-8]. These inferred organizational units were found to be associated with different regulatory elements, and therefore, distinct biological functions [9]. Another type of approaches, however, provided a probably more straightforward perspective. Dekker et al. pioneered a method called Chromosome Conformation Capture (3C) [10] to examine the physical and spatial interactions between specific loci. With 3C, researchers can directly detect the higher-order DNA loops, which at least partially elucidate the structural basis of certain organizational units with specific functions [11-14]. Nonetheless, the applications of 3C and its derivatives require pre-selected loci, which limit more global insights into genome organization [15].

Recently, a technology called Hi-C, a novel derivative of 3C coupled with massively parallel pair-ended sequencing, has been used to generate an unbiased genome-wide mapping of the DNA interactome [16]. From the analysis of Hi-C data, Botta et al. discovered that strong long-range genomic interactions could be maintained through the activity of the CCCTC-binding factor (CTCF) [17]. Another group demonstrated that distal genomic rearrangements in early replication domains are enriched with DNA interactions[18]. As Hi-C technology monitors higher-order DNA looping at the genome scale, this technology provides the opportunity to study the genome organization and also poses the challenges in the development of analytical methods. Although Lieberman-Aiden et al. employed Principal Component Analysis to segregate the whole genome into two compartments based on Hi-C data [16], attempts to explore the more detailed organization from Hi-C data are still lacking.

In this study, we thus propose a two-step strategy, titled Genome Segmentation from Intra-Chromosomal Associations (GeSICA), to investigate genome organization based on Hi-C data. We applied the method to Hi-C data in both the GM06990 and K562 cell lines. In the first step, GeSICA calculates a simple logged ratio to categorize the entire human genome into two different states. Regions in one of the states are significantly enriched with active genes and transcription factor binding sites (indicated as "plus states"), whereas regions in the other state are relatively less active (indicated as "minus states"). In the second step, we further segregated the plus-state regions into more detailed clusters by employing a Markov Clustering algorithm. These clusters are characterized by a relatively higher probability of DNA interactions inside rather than across clusters [19]. The insulator CTCF and one subunit of cohesin, namely, Rad21, were observed to be preferentially located in the boundaries between neighboring clusters, as were the proteins and histone marks related to transcription activities, including RNA polymerase II (Pol II), transcription initiation factor TFIID subunit 1 (Taf1) and H3K79me2. Taken together, these clues imply that the inferred clusters may achieve a finer and more detailed level in describing the features of genome organization.

## Results

### Dichotomization of human genome into two genomic states

**Figure 1:**
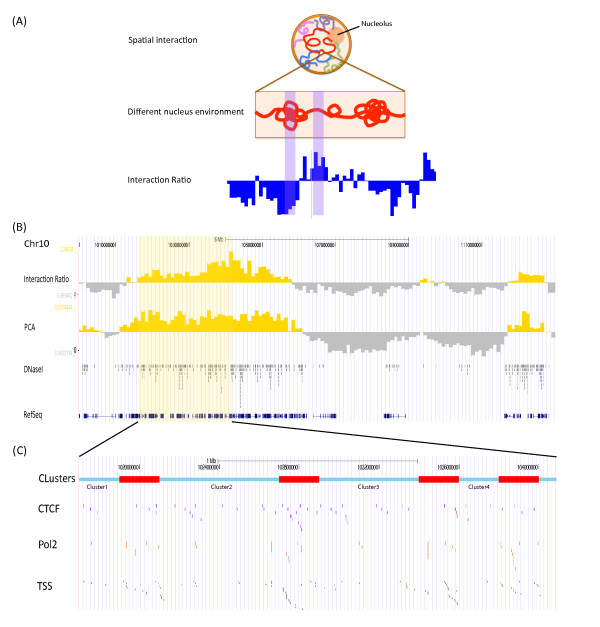
**Explanation and illustration of the "Interaction Ratio".** (**A**) An illustration of the theoretical assumption of interaction ratios is shown here. The first part is a description of the nucleus. Strings with different colors are chromosomes. The genome has open and compact chromatin, as shown in the middle section; in the open chromatin, more random short-range interactions are easier to detect by Hi-C experiment. And the spectrum-like interaction ratio profile is displayed in the last section. (**B**) In part of Chromosome X, visualization of the interaction ratios and first principal component signals is displayed using the UCSC Genome Browser for Hi-C data in the GM06990 cell line. The yellow portion of the signals represents the bins with plus (+) signals; the gray portions are bins with minus (-) signals for both interaction ratios and first principal components. The two signals are quite similar. The DNase I Hypersensitive Sites Peaks were acquired here as a reference of the open chromatin. The RefSeq genes are also included in this figure. (**C**) A close look of a region in Chromosome X, from position 101,000,000 - 104,000,000. The first track is a snapshot of the clusters and boundaries after Markov Clustering. Boundaries between neighboring clusters are highlighted as "red blocks". The binding sites of CTCF and Pol II and the locations of transcriptional start sites are displayed to show their levels of enrichments in boundaries.

GeSICA was applied to Hi-C data to dichotomize the human genome by introducing a simple parameter, the interaction ratio, to capture the structural characteristics of two different states. It is based on the following assumption: short-range random DNA interactions would be easier to detect in open chromatin environments than in more close ones (Figure 1A). The whole genome was first divided into bins with 100Kb as bin-size, and the interaction ratio was calculated for each bin as a log-transformed normalized ratio of short-range to long-range DNA interaction counts (see Materials and Methods for details). As the profile of the calculated interaction ratios displayed apparent boundaries between neighboring "plus signals" and "minus signals" (Figure 1B), we divided the genome into regions with two distinct states according to the sign of the interaction ratio: i.e. plus and minus state separately. After removing gaps in the assembled genome, approximately 40% of the genomic regions in human GM06990 cell line were assigned as plus state and 60% were assigned a minus state ( Additional file 1: Figure S 1A, Additional file 1: S 1C).

**Figure 2:**
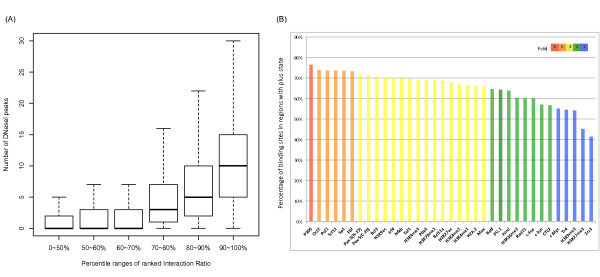
**Functional validation and exploration of regions with different interaction ratios and two genomic states in GM06990 cell line.** (**A**) Shown is the distribution of DNase I HS site numbers in bins grouped by different percentile ranges of ranked interaction ratios (0 ~ 50%, 50% ~ 60%, 60% ~ 70%, 70% ~ 80%, 80% ~ 90% and 90% ~ 100% are listed here). The numbers of the DNase I HS sites increased with the interaction ratio. (**B**) Displayed are percentages of different transcription factor binding sites and histone modification peaks located in plus-state region. Different colors indicated different scales of fold-enrichment of the binding sites in plus-state regions to the minus-state regions in GM06990 cell line. P300 had the highest proportion of binding sites in plus states, while Zzz3 and H3K27me3 had the lowest.

As the interaction ratio was designed to capture chromatin environment, we subsequently evaluated this parameter by comparing it to the degree of chromatin openness. In this evaluation, we used the number of DNase I hypersensitive (HS) sites within each genomic bin as a benchmark of the degree of chromatin openness. As shown in Figure 2A, the genomic bins were grouped based on the percentile ranges of the ranked interaction ratios, as calculated from Hi-C data in GM06990 cell line. There was a clear trend that the number of DNase I HS sites increased with the interaction ratios. Therefore, the interaction ratio calculated from Hi-C data can be regarded as an index that quantifies the degree of chromatin openness.

To extend our exploration of the properties of inferred plus and minus states, we employed dozens of transcription factor binding profiles and histone modification profiles to examine their distributions in genomic regions with different states (we define a region as a series of continuous bins with the same state). Compared with regions with the minus state, those regions with the plus state were dominantly enriched with a plethora of important transcription factors and histone modifications (all available through the ENCODE Project from UCSC Genome Browser [20]) in the GM06990 cell line. For 27 of the 35 transcription factors and histone marks, including P300, Sp1 and H3K4me3, over 60% of their ChIP-Seq peaks were located in regions with the plus state (Figure 2B). For each transcription factor, we also calculated the fold-enrichments of peak numbers in regions with the plus state to those with the minus state. Most were larger than 1.5, except for Zzz3 and H3K27me3. H3K27me3 is a typical repressive histone mark [21] and Zzz3 is the subunit of the Ada-Two-A-containing complex, which has been shown to be responsible for regulating the activity of non-histone targets and controlling mitotic progression, while accompanied by high levels of chromatin compaction [22]. In contrast to the plus state, regions with the minus state appeared to behave as functional deserts with a small fraction of transcription factor bindings or histone marks present. Altogether this evidence is consistent with the degree of chromatin openness between regions with plus and minus states. Similar results were obtained in the human K562 cell line ( Additional file 1: Figure S 2Additional file 1: Figure S 1B, Additional file 1: S 1D). These observations indicate that the regions with the plus state dominantly contain more interested information due to the fecundity of transcription factor binding and histone marks in the relatively open chromatin. Thus, the detailed organization of regions with the plus state became the focus of our subsequent analysis.

In a previous study, Lieberman-Aiden et al. employed principal component analysis (PCA) on the correlation matrix of DNA interactions to segregate the human genome into two compartments [16]. The value of the first principal component (PC) from their approach could also be taken to reflect the degree of chromatin openness ( Additional file 1: Figure S 3). Thus, the genome can be segmented into regions characterized as one of the two states according to the sign of first PC: which could also be called plus and minus state separately. In the GM06990 cell line, the segmentation results of the two approaches were largely similar, and 75.2% of genomic bins were inferred to have the same state (Figure 1B). Although both approaches achieved similar results, their basic assumptions are quite different. The approach of Lieberman-Aiden et al. was based on the assumption that the interaction vectors of genomic bins (with 100Kb or 1 Mb as bin-size) are more similar within one compartment than across two different compartments. As mentioned previously, however, our approach was based on the assumption that short-range random DNA interactions would be easier to detect in relatively open chromatin environments (Figure 1A). Overall, our approach provided a new perspective by which to interpret different states in the human genome from Hi-C data, which exhibits a performance similar to that of PCA.

### Detailed segmentation in regions with plus states

**Figure 3:**
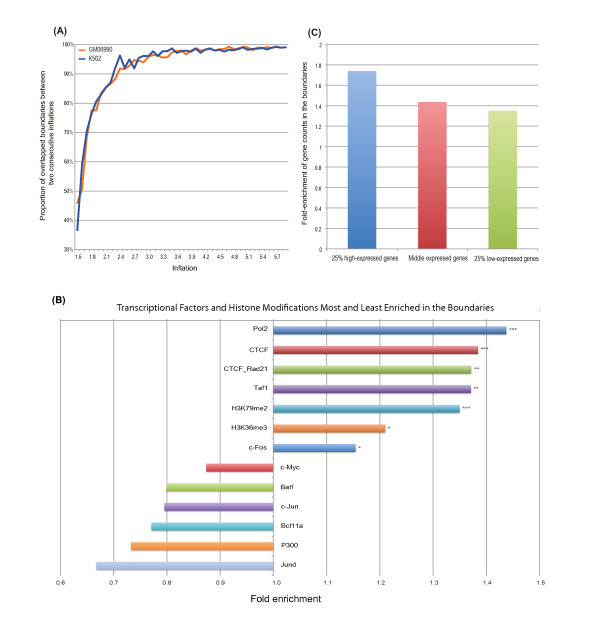
**Markov Clustering validation and features of the boundaries of the detailed segmentation.** (**A**) Displayed is stability of Markov Clustering result in two cell lines: the percentage of shared boundaries between two consecutive inflation parameters reached a plateau at inflation around 3.0. (**B**) Transcription factors and histone modifications most and least enriched in the boundaries between neighboring clusters are shown for the GM06990 cell line. Only clusters with at least one bin not in the cluster boundary were considered. The most enriched are the binding sites of PolII, CTCF, Taf1 and the co- binding sites of CTCF and Rad21. ***: adjusted p-value < 10^-40^, **: adjusted p-value < 10^-10^, *: 10^-10^ < adjusted p-value < 10^-3^. (**C**) Shown are the comparisons of the enrichment of 25% high-‒;expressed genes, 25% low-expressed genes and the rest of the genes in the boundaries of GM06990. The higher the gene expressions, the more enrichment there is in boundaries.

To further segment the plus-state regions into more delicate clusters, we first excluded DNA interactions with physical distances below an empirical threshold 20Kb, in order to diminish the potential influence from random DNA interactions [16]. Markov Cluster algorithm (MCL) was then applied to intra-chromosomal DNA interactions to group the genomic bins into clusters such that DNA interaction counts within clusters were greater than those across clusters [23]. The clustering results were influenced by parameter inflation; the number of clusters increased steadily as inflation became larger ( Additional file 1: Table S 1). Varying this parameter from 2.4 to 6 (with 0.1 as step), the clustering results from two consecutive inflations shared a large percentage of cluster boundaries, and the percentage reached a plateau at an inflation value of approximately 3.0 (Figure 3A). This finding indicated that the increase in cluster number with larger inflation values was mainly due to the splitting of existing clusters into smaller ones. In this study, we adopted 3.0 as the default inflation value for MCL. In the human GM06990 cell line, a total of 1,495 clusters were inferred, with an average cluster size of approximately seven genomic bins; only 129 contained distal bins ( Additional file 1: Figure S 4A-E). A detailed illustration of the clusters is shown in Figure 1C by focusing on the region around 2 Mb. The binding sites of CTCF and Pol II and locations of Transcription Starts Sites were also visible (another example is shown in Additional file 1: Figure S 5). The DNA interaction counts within inferred clusters were significantly larger than those across clusters (1.42 fold, p- value < 2.2x10^-16^), suggesting that inferred clusters are relatively structurally independent. Besides, we randomly sampled 1,000 interaction datasets by generating the same amount interactions for any given genomic physical distance as in the actual Hi-C dataset. The average fold of DNA interaction counts within clusters against those across clusters is 1.18 in the simulated datasets, which is lower than the observed value in the real data (1.42) ( Additional file 1: Figure S 6). This result to some extent reflected the reliability of our clustering result and the appropriateness of using Markov Clustering on Hi-C data.

To explore the organizational features of the inferred clusters, we next assessed the distribution of transcription factors binding and histone marks in the boundaries between adjacent clusters. The genomic bins inside clusters were adopted as the control group. To ensure that each cluster had at least one bin not in the cluster boundary, we excluded all the clusters failing to meet the criteria in the following analysis. Among all of the transcription factor binding and histone marks available in the GM06990 cell line (through the ENCODE Project using the UCSC Genome Browser) [20], the binding sites of insulator CTCF were significantly enriched in the boundaries (fold around 1.38; adjusted p-value < 10^-41^). As CTCF has been shown to isolate long-range enhancers by looping DNA into higher-order structures that consequently maintain genomic structure [24-26], the results suggest that inferred clusters might be potential organizational units with independent structure and function, as demarcated by the insulator CTCF (Figure 3B). Similar results were obtained in human K562 cell line ( Additional file 1: Figure S 7A). Previous studies proposed that CTCF, together with cohesin, serves as an insulator in certain genomic regions [27-31]. The binding sites of Rad21, a subunit of cohesin, were only enriched in the cluster boundaries in K562 cell line. As Rad21 has twice as many binding sites in the GM06990 cell line as in the K562 cell line, we further examined the enrichment of the co–binding sites of CTCF and Rad21 in cluster boundaries. We found that CTCF-Rad21 co-binding sites were significantly enriched in the cluster boundaries of both cell lines (Figure 3B Additional file 1: Figure S 7A).

In the GM06990 cell line, the transcriptional start sites (TSS) of genes were significantly enriched in the boundaries of inferred clusters (fold 1.48, binomial test p-value < 2.2x10^-16^). In addition, the level of gene expression in the cluster boundaries was also higher than those within clusters. The top 25% of highly expressed genes had the highest fold-enrichment of gene number on boundaries inside clusters (1.74) compared with the middle 50% of genes (1.44) and the bottom 25% of genes (1.35) (Figure 3C Additional file 1: Figure S 7B). This result agrees with the one suggested in [32], which suggested the boundary functions of active TSSs. Consistent with the above results, several transcription process-related profiles were also enriched in the boundaries, including the ChIP-seq peaks of Pol II, Taf1, and H3K79me2 [33] (Figure 3B Additional file 1: Figure S 7A). Similar results were also observed in the K562 cell line ( Additional file 1: Figure S 7A-B). These observations indicated that the inferred cluster boundaries might be the potential spots that harbor hypothesized transcription factory compounded with transcription-related factors, and also suggested the potential insulator-like role of transcription factory or Pol II in humans, which may be similar to those reported in Drosophila [34,35].

### Dynamics of segmentation in GM06990 and K562 cell lines

**Figure 4:**
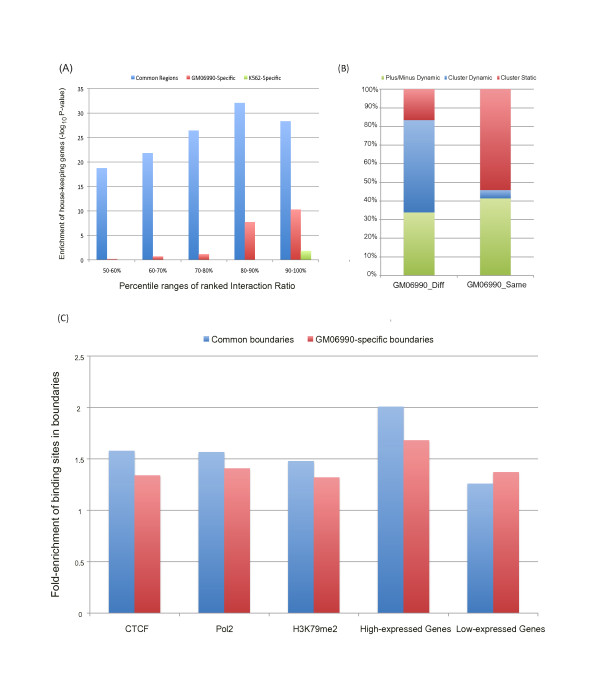
**Dynamic analysis of two genomic states and detailed segmentations.** (**A**) Housekeeping genes enrichments in cell-type-common bins, GM-specific bins and K562-specific bins are compared under different percentile ranges of ranked interaction ratios (50-60%, 60%-70%, 70%-80%, 80%-90%, 90%-100%). The Y-axis is the enrichment score (-log10(p-value), from the Binomial test) for each percentile range. Bins with high interaction ratios in both cell lines are more enriched with housekeeping genes. (**B**) Cluster dynamics analysis of each pair of neighboring bins: the dynamics of the Diff category (two bins belonging to different clusters) and the Same category (two bins belonging to the same cluster) are examined. The fractions of different dynamic activities are displayed with different colors. Green: original category in GM06990 cell line turns into Minus category (two bins with at least one in minus states) in K562 cell line. Blue: the category alters between Diff and Same from GM06990 to K562 cell line. Red: the category does not change from GM06990 to K562 cell line. In GM06990 cell line, only 20% of the pairs in the Diff category remained to be in Diff in K562. Approximately 50% in the Diff category became pairs in the Same category. More than half of pairs of adjacent bins in the Same category remained as the Same in K562. (**C**) The fold-enrichment of CTCF, Pol II, H3K79me2, top 25% high-expressed genes, and top 25% low-expressed genes in the boundaries of the plus states in GM06990 cell line. Blue: Cell-type common boundaries. Red: GM06990-specific boundaries. The binding sites of CTCF and Pol II and the peaks of H3K79me2 are more enriched in cell-type-common boundaries than in GM06990-specific boundaries. A similar trend can be observed in high-expressed genes, whereas an opposite trend is shown in low-expressed genes.

We further examined the dynamics of genome organization between the GM06990 and K562 cell lines. Genomic bins were grouped based on the percentile ranges (10% as interval) of the ranked interaction ratios calculated from Hi-C data in each cell line separately. For each percentile range (e.g. 90-100%), genomic bins with interaction ratios in this range from both cell lines were regarded as cell-type-common bins, while those with ratios in this range in only one cell line were cell-type-specific bins. As shown in Figure 4A, cell-type-common bins were highly enriched with housekeeping genes across different percentile ranges. The cell-type-specific bins, however, had much less significant enrichment or even non-significant enrichment of those genes. We also checked the enrichment of gene ontology (GO) categories or genes in cell-type-common and cell-type-specific bins separately. Genes were assigned to bins according to their transcriptional start sites. Those genes in the K562-specific bins of the highest two percentile ranges (90-100% and 80-90%) were enriched with "chemokine activity" (adjusted p-value: 1.2x10^-4^ and 1.4x10^-5^) and "chemokine receptor binding" (adjust p-value: 1.7x10^-4^ and 2.6x10^-5^), whereas no GO categories were found to enrich for other cell-type-common or cell-type-specific bins. This result indicates that there might be a direct relationship between chemokine receptors and leukemia, as suggested by several other recent studies [36-38].

To capture the characteristics of dynamics of detailed organization, we compared the inferred clusters in the plus-state regions of the GM06990 and K562 cell lines. For each cell line, every pair of adjacent bins in the inferred clusters was assigned a category to indicate one of the following two types of relationships: two bins belonging to the same clusters (Same) and to different clusters (Diff). In the GM06990 cell line, only 20% of the pairs in the Diff category remained Diff in K562, while approximately 50% in the Diff category became pairs in the Same category. The remaining pairs contained at least one bin with the minus state in K562 (indicated as the Minus category). In contrast, more than half of the pairs of adjacent bins in the Same category stayed Same in K562 (Figure 4B). These observations imply that the boundaries between inferred clusters are notably dynamic.

To investigate whether some important functional elements were enriched in the boundaries between adjacent clusters in a cell-type-specific manner, we classified the boundaries in the GM06990 cell line into two types: common boundaries (those that were also cluster boundaries in K562 cell line) and GM-specific boundaries (those within clusters or with the minus state in K562). In total, 204 bins were identified as common boundaries, and 938 bins were determined to be GM-specific boundaries. We found that the binding sites of CTCF and Pol II in the GM06990 cell line were more enriched in the common boundaries than in the GM-specific ones (Figure 4C). A similar trend was observed for the top 25% highly expressed genes in contrast to the bottom 25% genes (Figure 4C). The above phenomenon was observed regardless of the cell line ( Additional file 1: Figure S 8).

Next, we examined the relationship between the differential expression of genes and the dynamics of detailed organizations. Genomic bins with plus states in both cell lines were ranked by the proportion of differentially expressed genes between the GM06990 and K562 cell lines, while the list of differentially expressed genes was calculated using Limma software package [39]. Each genomic bin with the plus state in both cell lines belongs to two inferred clusters in the two studied cell lines, and a Jaccard index was introduced to evaluate the similarity between the two clusters. Here we selected the top 10% and bottom 10% ranked bins. As shown in Additional file 1: Figure S 9, there was a trend that the higher the proportion of differentially expressed genes, the smaller the Jaccard Index, i.e., the less similarity between the clusters to which bins in the two cell lines belonged. The results suggested that the differentially expressed genes were potentially associated with the dynamics of genome organization around them.

## Discussions

Despite some interesting insights into the inferred genome organization that were raised during this investigation, our approach still faces analytical challenges and limitations. First, due to the current sequencing depth of Hi-C experiments, it is difficult to further narrow bin size (i.e. organizational resolution). Therefore we cannot observe more detailed organization (e.g. at several or dozens of Kbs in length) that might be more relevant to specific functions.Secondly, the use of different restriction enzymes in Hi-C experiments may have caused systematic biases. To evaluate the potential bias from enzymes on inferred genome organization, GeSICA was applied to Hi-C data in the GM06990 cell line using either HindIII or NcoI as restriction enzymes separately. The calculated interaction ratios from each dataset were notably similar to those from the combined datasets ( Additional file 1: Figure S 10A, Additional file 1; S 10B). From each dataset, we also observed the enrichment of the binding sites of CTCF, Pol II and Taf1 in the inferred cluster boundaries ( Additional file 1: Figure S 11A, Additional file 1: S 11B). From our analysis, the inferred genome organization from Hi-C datasets, as determined using different enzymes, was generally consistent to each other. Last, GeSICA is a general method of genome segmentation from Hi-C data that does not consider the rearrangement of the cancer genome, although genome rearrangement might affect inferred organization.

## Conclusions

We introduced a two-step strategy, GeSICA, which can be used to investigate the genome organization based on Hi-C data. The first step was based on the assumption that random short-range DNA interactions would be easier to detect in open chromatin environments, and that the calculated interaction ratio could be regarded as an index that quantifies the degree of chromatin openness. The second step was designed on structural features of chromatin organization: the levels of DNA interactions within clusters are greater than those across clusters, and the inferred clusters may be potential organizational units with independent structures and functions. In the foreseeable future, we expect numerous studies to generate unbiased DNA interactome data with improved resolution that will enhance the credibility and efficacy of genome organization characterizations.

## Materials and methods

### Interaction ratio

"Genomic bins" are defined as non-overlapping genomic intervals of a certain length. Several continuous bins comprise a genomic region. In this study, the default bin-size was set as 100Kb. One end of each paired-end read was considered to be in a bin if its starting position fell within the interval of that bin. For each pair of bins, the number of paired-end tags was set as the absolute interaction value (IV).

The absolute interaction value was then scaled by the distance between two bins as demonstrated by the following formula: lc is the total number of bins in the specific chromosome, m and n are the indices for any possible pairwise bins on a certain chromosome and p and q are any two bins with the same distance as that between m and n:

(1) $$NormIV_{\mathrm{mn}}=IV_{\mathrm{mn}}/\left(\sum_{p-q=m-n}IV_{\mathrm{pq}}/\left(l_{c}-p+q\right)\right)$$

Next, the interaction ratio (R) was computed for each bin using the following formula:

(2) $$R_{i}=log\left(\sum_{j=i-d}^{i+d}NormIV_{\mathrm{ij}}/\sum_{k=i+d+1}^{l_{c}}NormIV_{\mathrm{ik}}\right)$$

In this formula, d was set as the parameter to determine the distal interactions. For the sake of convenience in discretizing the genomic state, we used the logged form of this ratio to generate a spectrum like profile.

### Markov Clustering

Markov Clustering [23,40] is a clustering algorithm designed for the natural partitioning of weighted graphs. An intuitive perspective of Markov Clustering is to detect the clusters in which random walks would infrequently lead to another one. The whole process is deterministic. Markov Clustering firstly transforms the input adjacency list into a stochastic "Markov" matrix. This matrix portrays the transition probabilities between all pairs of bins. Markov Clustering then simulates random walks in a graph by two major steps named expansion and inflation. Expansion is calculated by taking the stochastic matrix squaring (or n^th^ powering) to calculate the probability of a random walk of length n. The probabilities between bins in the same cluster will be higher than that across different clusters. Furthermore, to aggrandize this effect, an inflation step is raised to take the entry wise Hadamard power of a matrix and then followed by a normalization step to turn the new matrix back into a stochastic or Markov matrix again. Finally, clusters are detected by repeating and alternating expansion and inflation until convergence is obtained, i.e., the probability between the final clusters is less than the given tolerance.

### Software implementation

GeSICA is implemented in Python and freely distributed with an open source Artistic License at [41]. The following parameters should be used: -f for the Hi-C raw file format after reads mapping; -r for the desired resolution of the result; -d for the distance to distinguish short-range and long-range interactions; -c for filtering the interactions below a certain distance "c"; and all parameters necessary for Markov Clustering. The output files of GeSICA include three different types of files: an interaction-ratio wiggle file (-wig), a bed file for each bin with the corresponding state and cluster information (-bed) and a cluster file of the bins that each cluster contains (-cluster). GeSICA can be run from the command line and is available for Linux, UNIX and Mac OS. Additional file 1: Figure S 12 illustrates the workflow of GeSICA.

### Datasets used in this paper

The Hi-C experimental data and the result of PCA for the GM06990 and K562 cell lines from Lieberman-Aiden et al. can be obtained from the GEO database with accession No. GSE18199 [16] and from the Hi-C data browser [42]. The gene expression data of the GM06990 and K562 cell line can be downloaded separately from the GEO database with accession No. GSE14083 [43] and GSE12056 [44]. The list of differentially expressed genes were calculated using the Limma software package [39]. All of the ENCODE transcription factors binding sites, histone modifications, and DNaseI hypersensitive sites data can be accessed through the UCSC Genome Browser [20,45,46]. The genome assembly used in this work is hg18. If data from the GM06990 cell line was found not to exist, related data from the GM12878 cell line was employed instead, due to the similarity between these two cell lines [16]. The peak files of these data, which are available in both the hg18 and hg19 genome assemblies, were collected, and liftover was applied to transform the peak files that only existed in hg19 back to hg18. The list of human housekeeping genes was derived from [47], which reported 575 housekeeping genes in total.

## Abbreviations

Chromosome Conformation Capture, 3C; GeSICA, Genome segmentation from intra-chromosomal associations; CTCF, CCCTC-binding factor; Pol II, RNA Polymerase II; Taf1, Transcription initiation factor TFIID subunit 1; H3K9me1, Mono-Methylated Histone H3 at Lysine 9; H3K9me3, Tri-Methylated Histone H3 at Lysine 9; H3K27me3, Tri-Methylated Histone H3 at Lysine 27; H3K79me2, Di-Methylated Histone H3 at Lysine 79.

## Competing interests

The authors declare that they have no competing interests.

## Authors’ contributions

LL and YZ conceived of the project under the direction of YZ, JF, and JY. LL, YZ and YZ wrote the manuscript. LL, YZ, and NZ designed, wrote and implemented the software package. All of the authors participated in the discussions and contributed to the analysis of results throughout the project. All authors read and approved the final manuscript.

## Supplementary Material

Additional File 1 **Figures S1-S12 and Table S1.** This file contains supplementary figures S1-S12 and Table S1 corresponding to a summary of the results of Markov Clustering. Click here for file
