# Supplementary material for: GeSICA: Genome segmentation from intra-chromosomal associations
Source: BMC Genomics. 2012 May 4;13:164. doi: 10.1186/1471-2164-13-164 (PMC3460730; doi:10.1186/1471-2164-13-164)
Supplement: Additional File 1 — Figures S1-S12 and Table S1. This file contains supplementary figures S1-S12 and Table S1 corresponding to a summary of the results of Markov Clustering. [file 1471-2164-13-164-S1.pdf]

Figure S1. Size distribution of the regions only containing bins with the same state and distribution of Interaction Ratio

Shown are the distributions of the size of the regions only containing at least two bins with the same plus/minus state and distribution of Interaction Ratio, in (A), (C) GM06990 cell line and (B), (D) K562 cell line respectively.

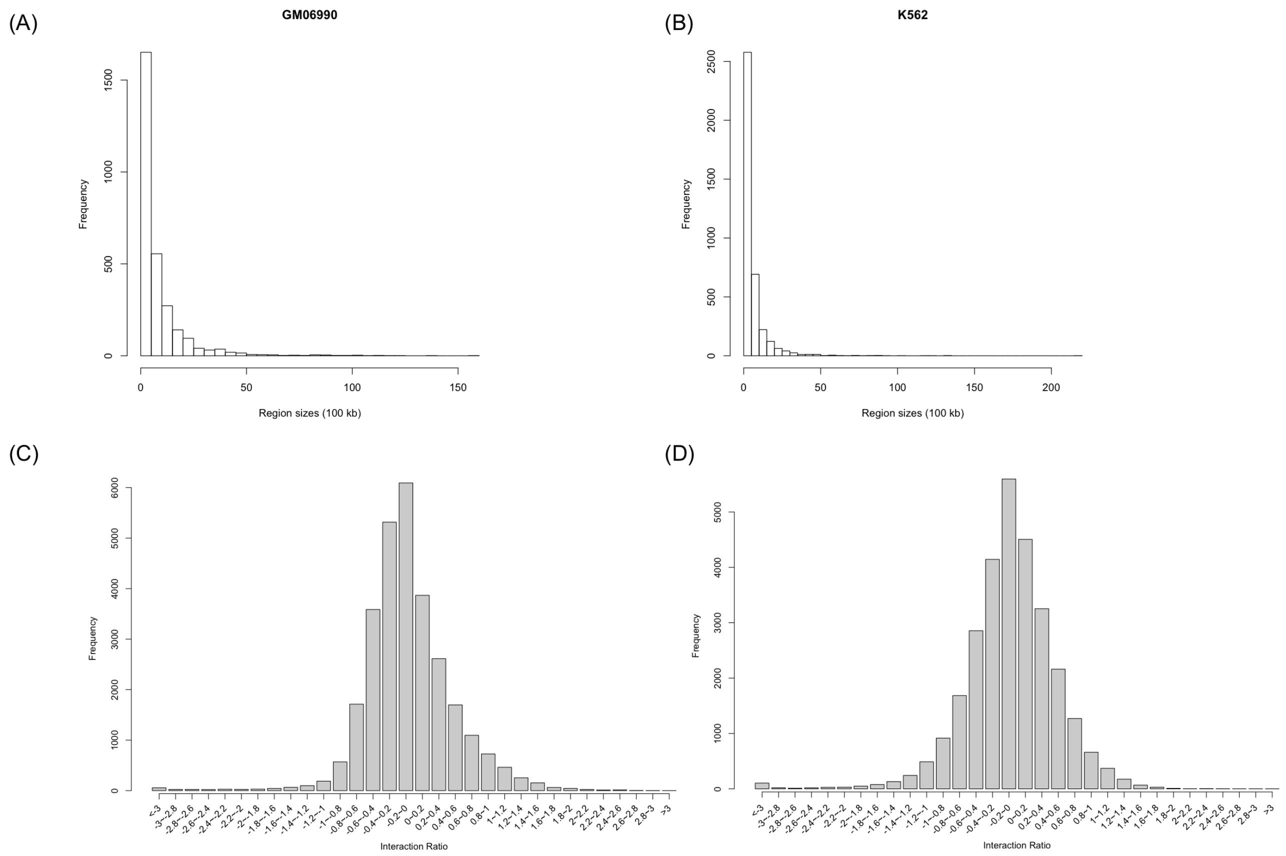

Figure S2. The functional validation and exploration of regions with different interaction ratios and two genomic states in K562 cell line

(A) Shown is the distribution of DNase I HS site numbers in bins grouped by different percentile ranges of ranked interaction ratios (0~50%, 50%~60%, 60%~70%, 70%~80%, 80%~90% and 90%~100% are listed here). The numbers of the DNase I HS sites increased with the interaction ratio. (B) Displayed are percentages of different transcription factor binding sites and histone modification peaks located in plus-state region. Different colors indicated different scales of fold-enrichment of the binding sites in plus-state regions to the minus-state regions in K562 cell line. TFIIIC had the highest proportion of binding sites in plus states, while H3K9me3, H3K27me3, and H3K9me1 had the lowest.

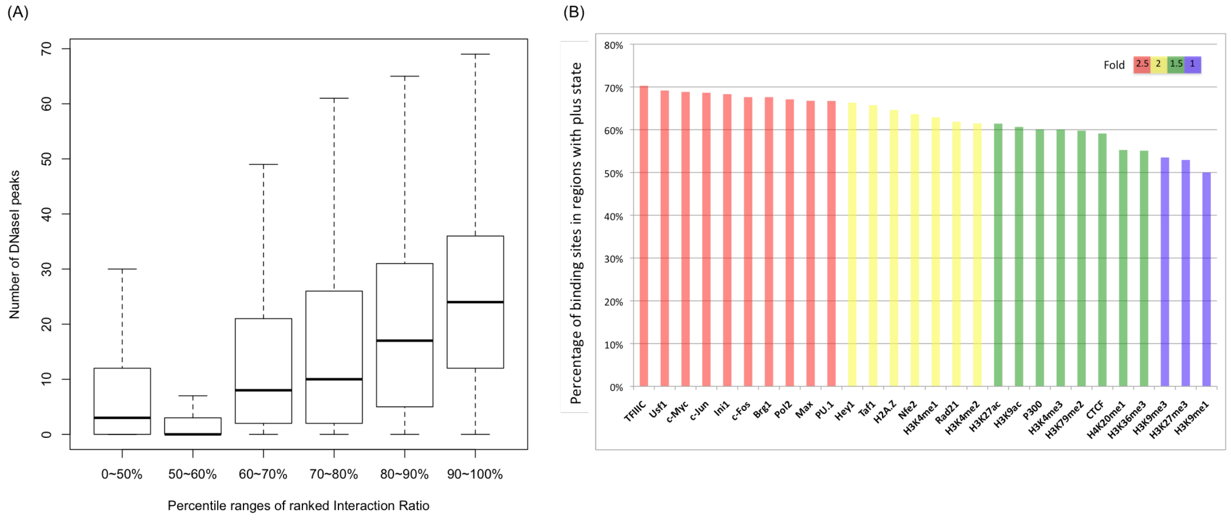

Figure S3. PCA versus DNaseI peaks

Shown is distribution of DNase I HS site numbers in bins with different percentile ranges of ranked first principal components (0~50%, 50%~60%, 60%~70%, 70%~80%, 80%~90% and 90%~100% are listed here). The numbers of the DNase I HS sites increased with the interaction ratio.

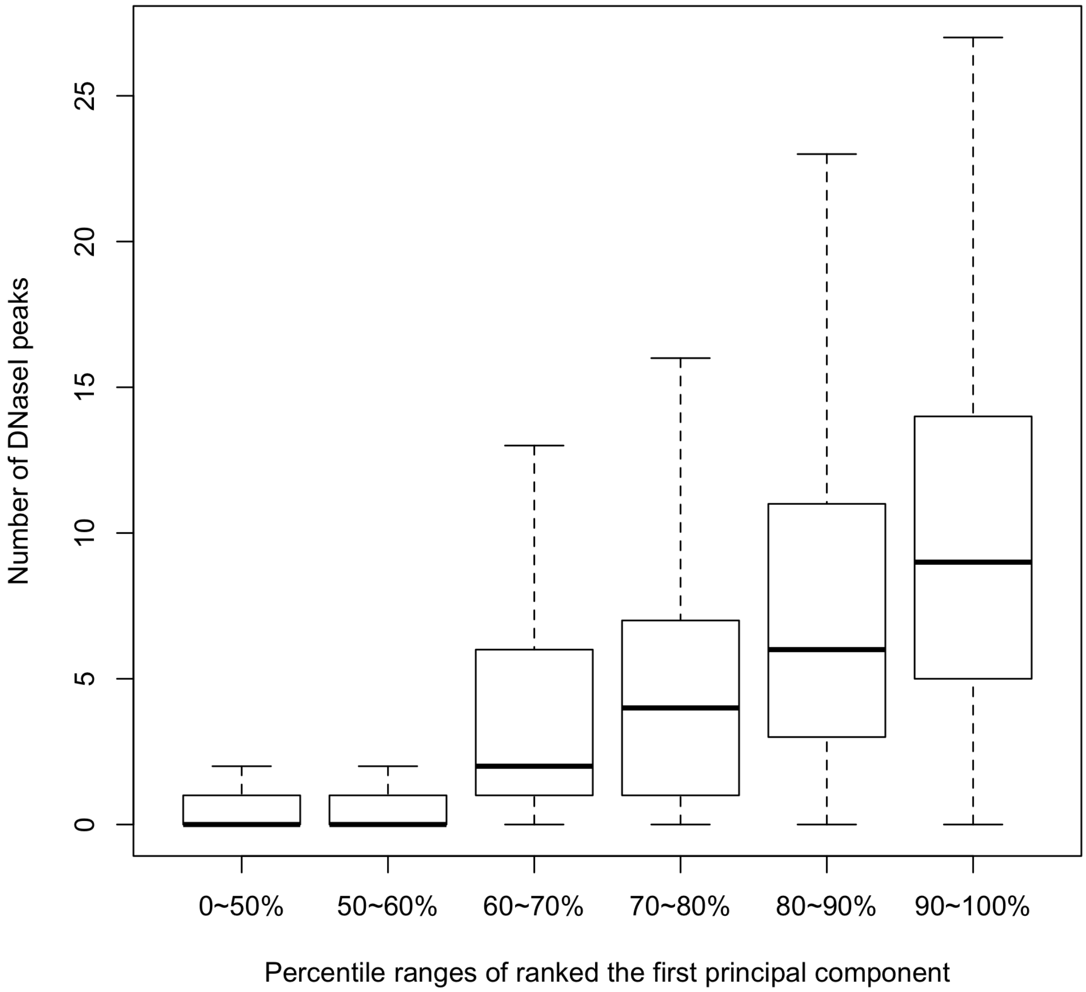

Figure S4. Summary of the results of Markov Clustering

(A) Shown are percentages of clusters with continuous bins and with distal bins in GM06990 and K562 cell lines. (B) – (E) The distribution of cluster lengths. (B) The clusters with continuous bins in GM06990 cell line. (C) The clusters with distal bins in GM06990 cell line. (D) The clusters with continuous bins in K562 cell line. (E) The clusters with distal bins in K562 cell line.

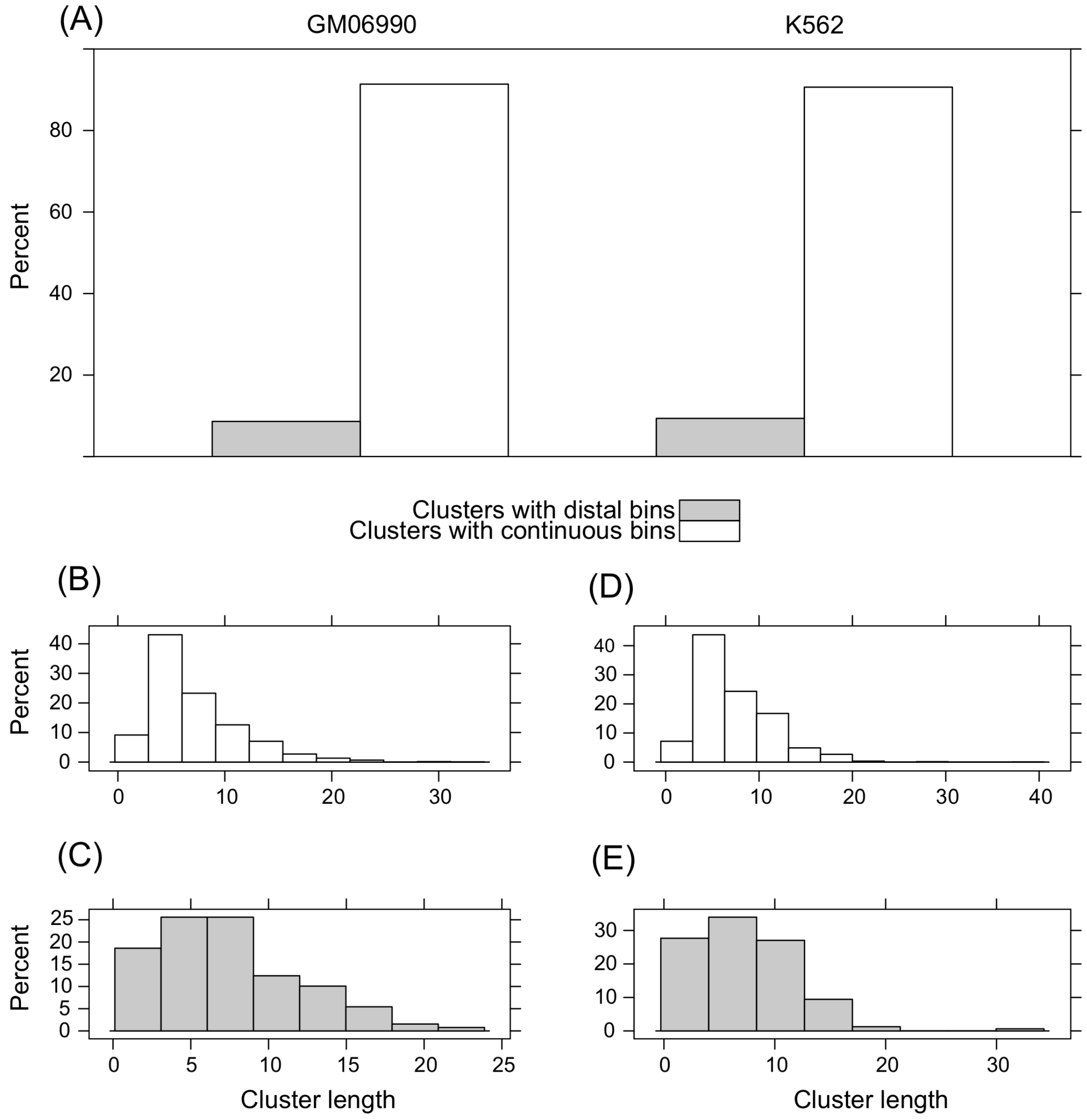

Figure S5. Illustration of the result of Markov Clustering

Displayed is a region in Chromosome I, from position 40,500,000 – 46,500,000. The first track is one snapshot of the clusters and boundaries after Markov Clustering. Boundaries between neighboring clusters are highlighted as “red blocks”. The binding sites of CTCF and Pol II and the locations of Transcriptional Start Sites are displayed to show their enrichments in the boundaries.

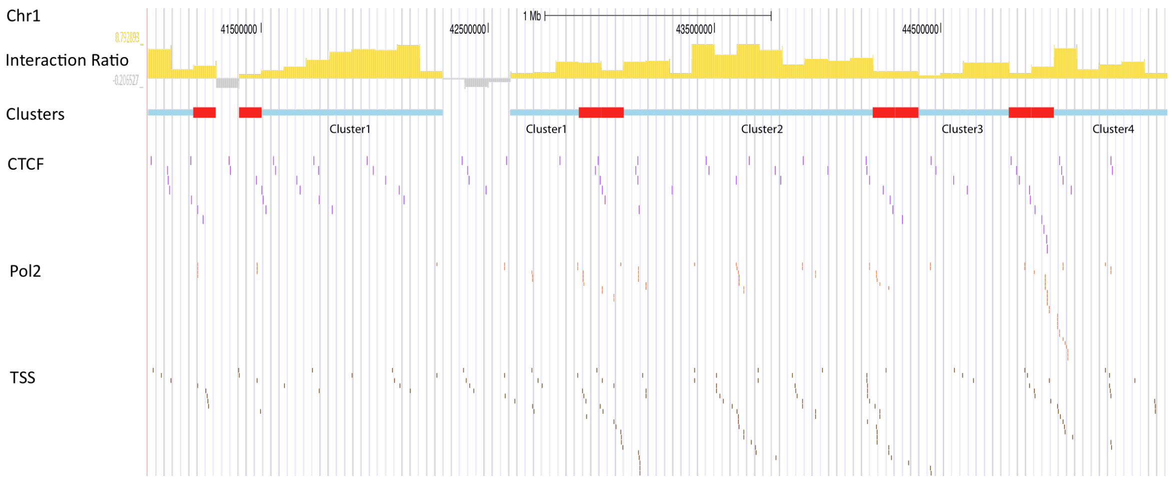

Figure S6. Distribution of fold-enrichments between interaction counts within clusters and across clusters from 1,000 simulated datasets

Displayed is the histogram for the fold-enrichments between interaction counts within clusters and across clusters from 1,000 simulated datasets. The simulated datasets have the same amount of interactions for any given genomic physical distance as the real Hi-C dataset. The average fold is 1.18.

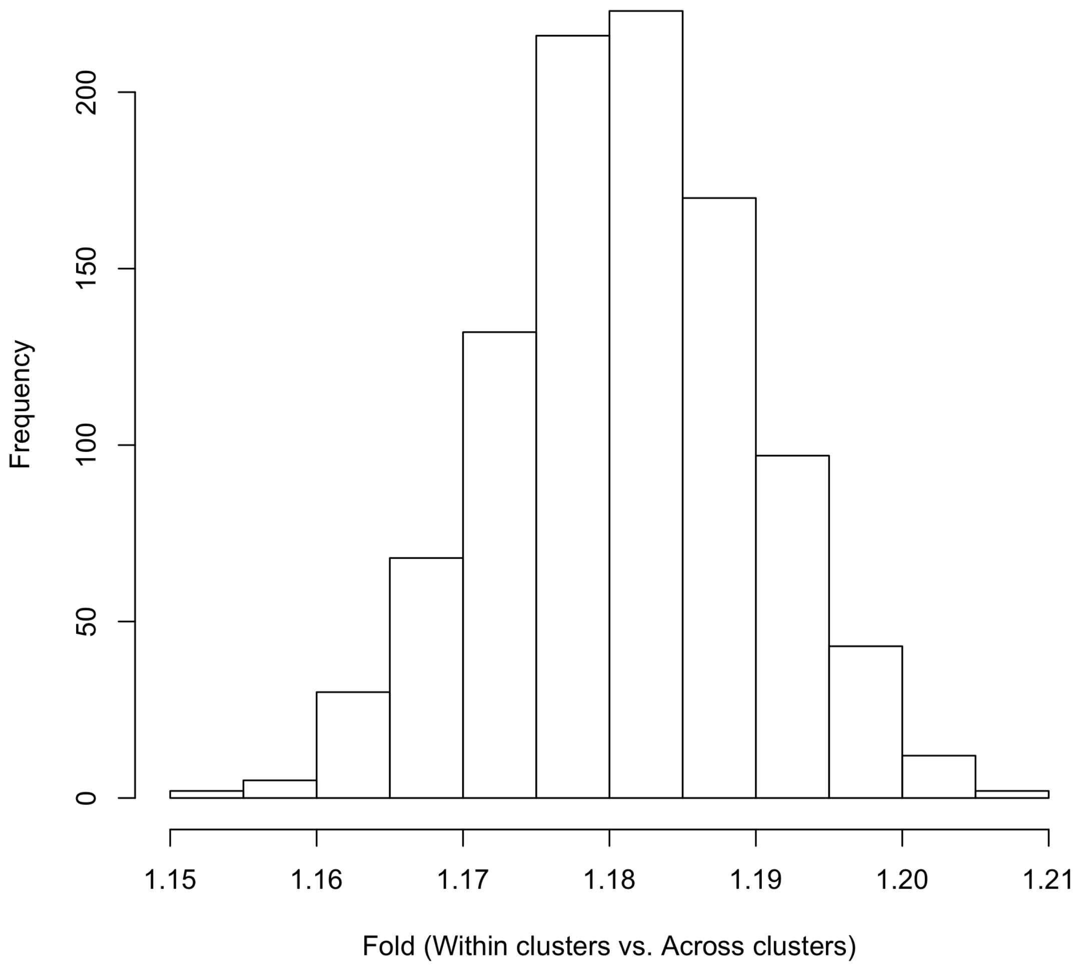

Figure S7. Transcription factor binding sites, histone modifications and genes enrichments in the boundaries between two neighboring clusters in K562 cell line

(A) Transcription factors and histone modifications most and least enriched in the boundaries between neighboring clusters are shown for the K562 cell line. Only clusters with at least one bin not in the cluster boundary were considered. The most enriched are the binding sites of TFIIIC and Pol II. ****: adjusted p-value < 10^{-90}, ***: adjusted p-value < 10^{-45}, **: adjusted p-value < 10^{-20}, *: 10^{-20}< adjusted p-value < 10^{-10}. (B) Shown are the comparisons of the enrichment of 25% highly expressed genes, 25% lowly expressed genes and the rest of the genes in the boundaries of K562 cell line. The higher the gene expressions are, the more enrichment in boundaries.

(A)

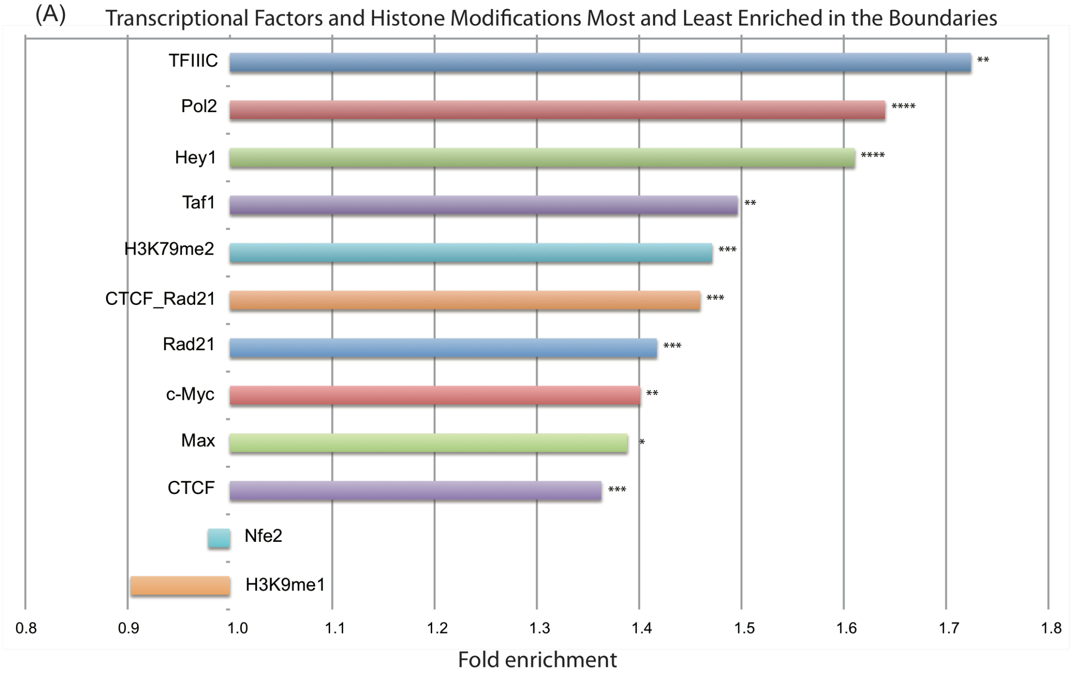
Transcriptional Factors and Histone Modifications Most and Least Enriched in the Boundaries
TFIIIC
**
Pol2
****
Hey1
****
Taf1
**
H3K79me2
***
CTCF_Rad21
***
Rad21
***
c-Myc
**
Max
*
CTCF
***
Nfe2
H3K9me1
0.8
0.9
1.0
1.1
1.2
1.3
1.4
1.5
1.6
1.7
1.8
Fold enrichment

(B)

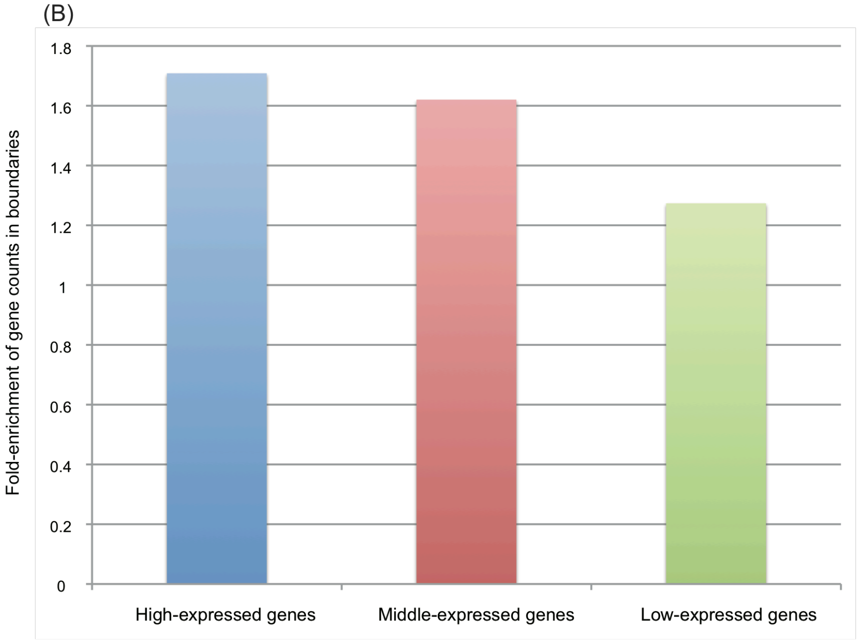
Fold-enrichment of gene counts in boundaries
1.8
1.6
1.4
1.2
1
0.8
0.6
0.4
0.2
0
High-expressed genes
Middle-expressed genes
Low-expressed genes

Figure S8. Dynamic boundaries in K562 cell line

The fold-enrichment of CTCF, Pol II, H3K79me2, top 25% high-expressed genes, and top 25% low-expressed genes in the boundaries of the plus states in K562 cell line. Blue: Cell-type common boundaries. Red: K562-specific boundaries. The binding sites of CTCF and Pol II and the peaks of H3K79me2 are more enriched in cell-type-common boundaries than in K562-specific boundaries. A similar trend can be observed in high-expressed genes, whereas an opposite trend is shown in low-expressed genes.

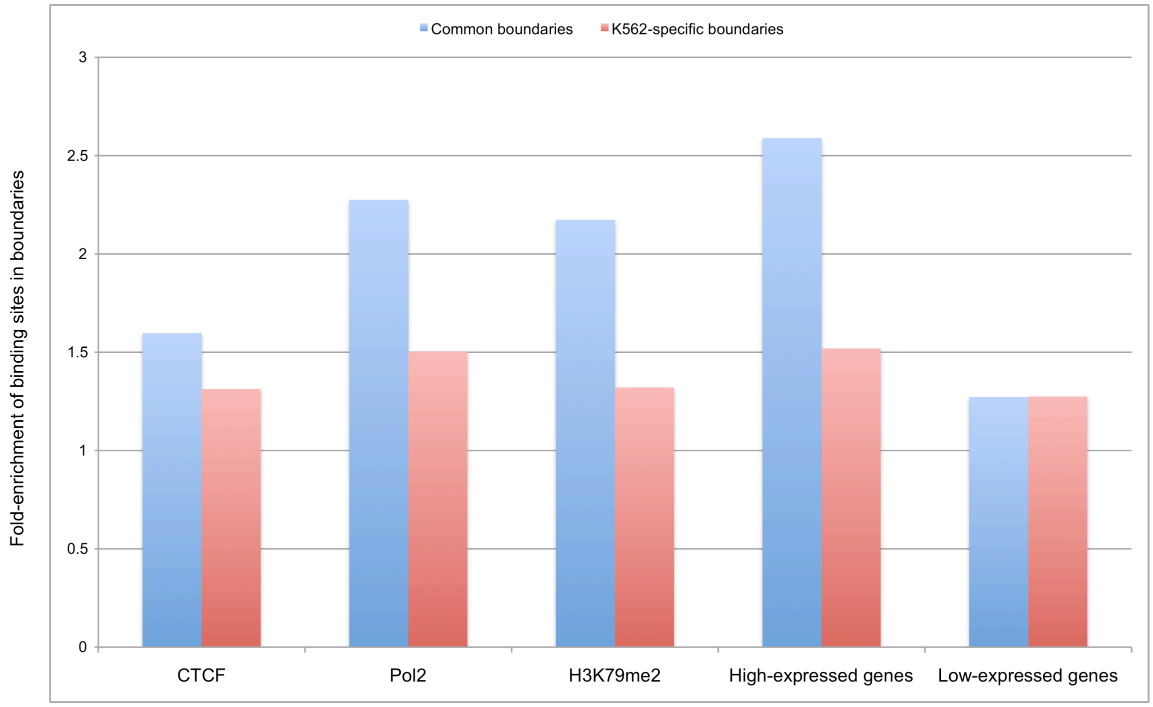

Figure S9. Dynamic clusters and differential gene expressions

The distributions of Jaccard Index in two groups are shown in this figure. One group is the bins with 10% highest proportion of differential expressed genes, and the other one is the bins with 10% lowest proportion of differential expressed genes. We calculated the Jaccard Index for each bin, comparing the similarity between the clusters that this bin belonged to in the two cell lines respectively. *: bins with the designated proportion of differential expressed genes

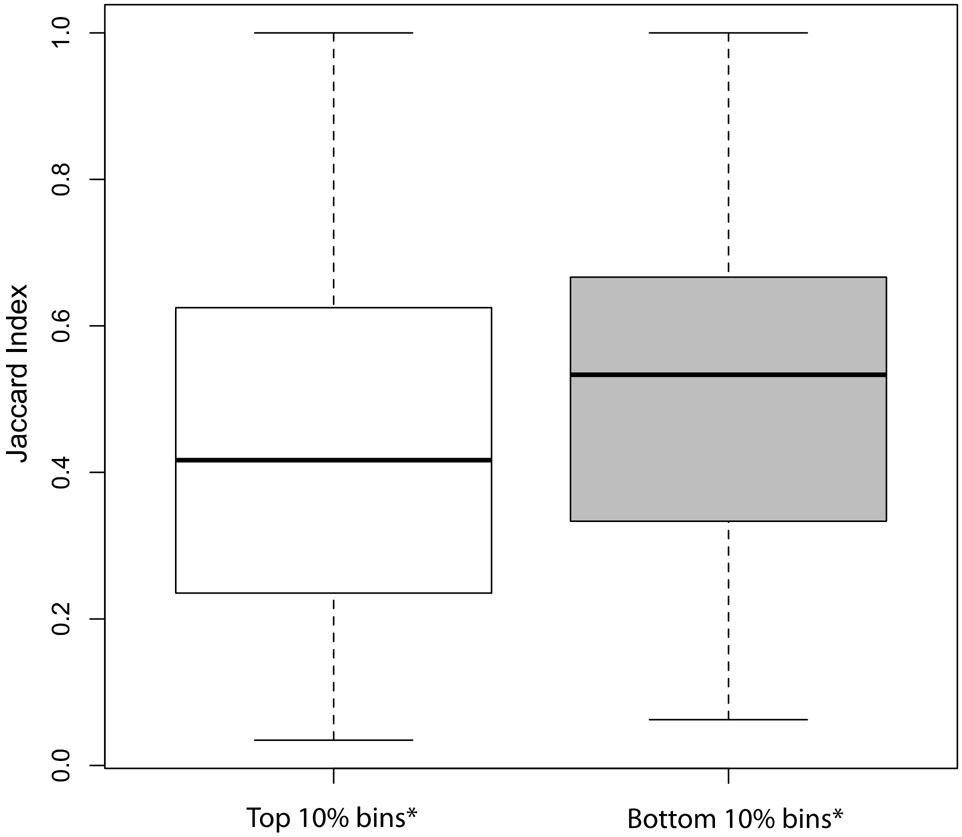

Figure S10. Results of HindIII GM06990 datasets

(A) Displayed are percentages of different transcription factor binding sites and histone modification peaks located in plus-state region. Different colors indicated different scales of fold-enrichment of the binding sites in plus-state regions to the minus-state regions in HindIII dataset of GM06990 cell line. (B) Transcription factors and histone modifications most and least enriched in the boundaries between neighboring clusters are shown for the GM06990 HindIII dataset. Only clusters with at least one bin not in the cluster boundary were considered. The most enriched are the binding sites of CTCF and Pol II and the co-binding sites between CTCF and Rad21. ***: adjusted p-value $< 10^{-30}$, **: adjusted p-value $< 10^{-10}$, *: $10^{-10} <$ adjusted p-value $< 10^{-3}$.

(A)

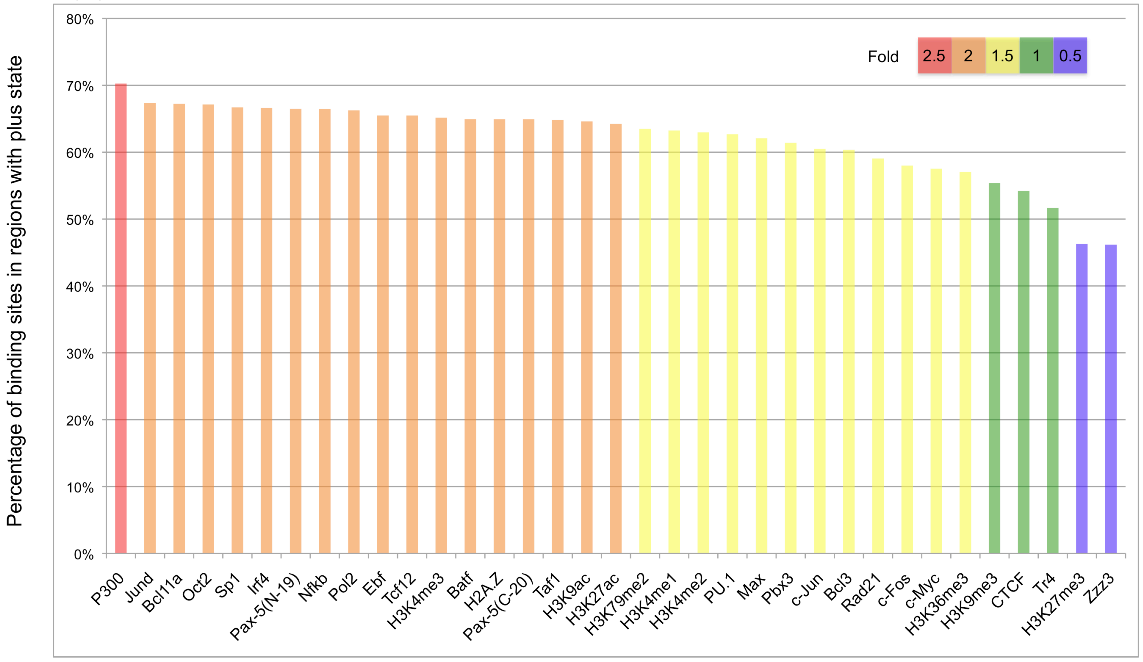

(B) Transcriptional Factors and Histone Modifications Most and Least Enriched in the Boundaries

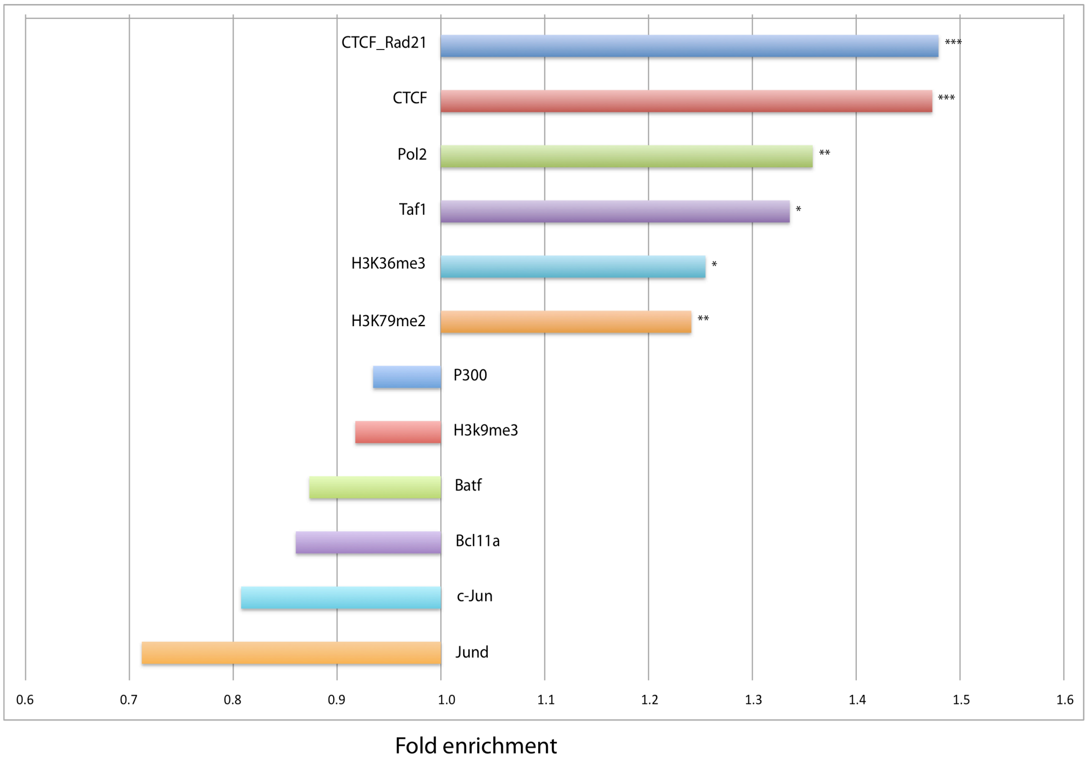

Figure S11. Results of NcoI GM06990 datasets

(A) Displayed are percentages of different transcription factor binding sites and histone modification peaks located in plus-state region. Different colors indicated different scales of fold-enrichment of the binding sites in plus-state regions to the minus-state regions in NcoI dataset of GM06990 cell line. (B) Transcription factors and histone modifications most and least enriched in the boundaries between neighboring clusters are shown for GM06990 NcoI dataset. Only clusters with at least one bin not in the cluster boundary were considered. The most enriched are the binding sites of Pol II and CTCF, and H3K79me2. ***: adjusted p-value $< 10^{-30}$, **: adjusted p-value $< 10^{-10}$, *: $10^{-10} <$ adjusted p-value $< 10^{-3}$.

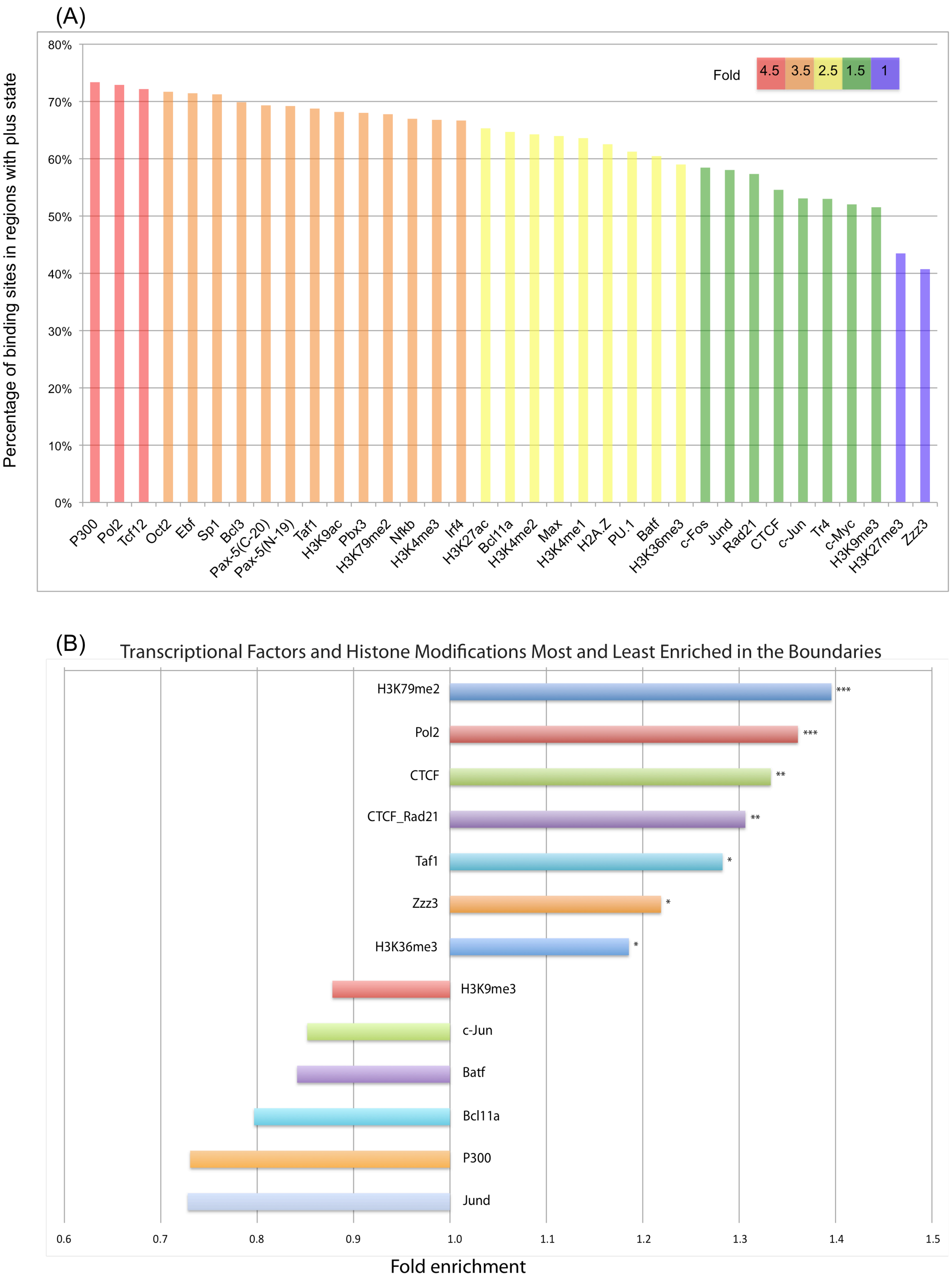
(A)
Percentage of binding sites in regions with plus state
80%
70%
60%
50%
40%
30%
20%
10%
0%
Fold
4.5
3.5
2.5
1.5
1
P300
Pol2
Tcf12
Oct2
Ebf
Sp1
Bcl3
Pax-5(C-20)
Pax-5(N-19)
Taf1
H3K9ac
Pbx3
H3K79me2
Nfkb
H3K4me3
Irf4
H3K27ac
Bcl11a
H3K4me2
Max
H3K4me1
H2A.Z
PU.1
Batf
H3K36me3
c-Fos
Jund
Rad21
CTCF
c-Jun
Tr4
c-Myc
H3K9me3
H3K27me3
Zzz3
(B)
Transcriptional Factors and Histone Modifications Most and Least Enriched in the Boundaries
H3K79me2
***
Pol2
***
CTCF
**
CTCF_Rad21
**
Taf1
*
Zzz3
*
H3K36me3
*
H3K9me3
c-Jun
Batf
Bcl11a
P300
Jund
0.6
0.7
0.8
0.9
1.0
1.1
1.2
1.3
1.4
1.5
Fold enrichment

Figure S12. Workflow chart of GeSICA

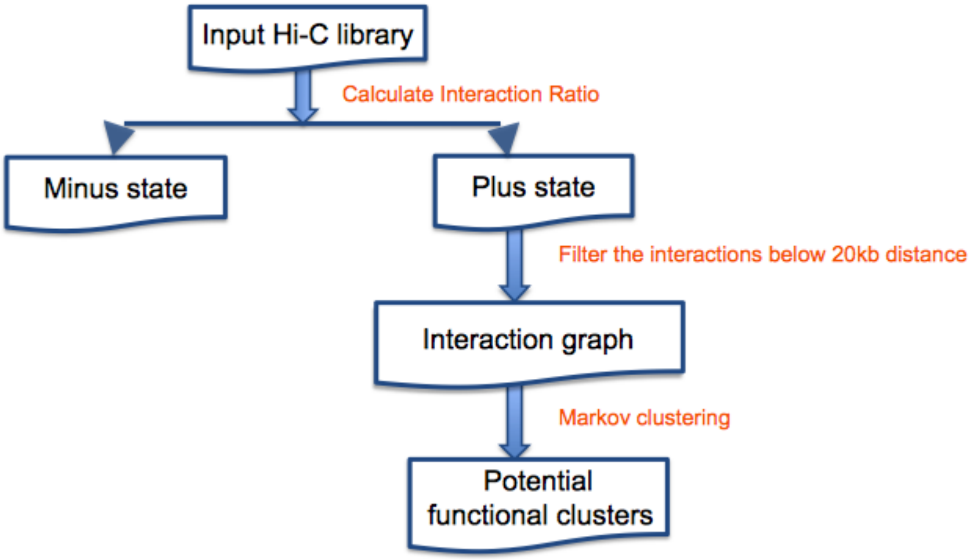

Table S1. Summary of the results of Markov Clustering

	GM06990					K562				
	Continuous Clusters		Clusters with distal regions		Intra/inter cluster interaction fold*	Continuous Clusters		Clusters with distal regions		Intra/inter Cluster interaction fold*
Inflation	No.	Ave-length	No.	Ave-length		No.	Ave-length	No.	Ave-length	
1.5	344	22.8	86	32.5	1.49	405	24.0	84	30.1	1.33
1.6	491	17.7	88	22.5	1.38	529	18.8	109	21.9	1.33
1.7	618	15.0	81	17.2	1.36	669	16.0	105	16.2	1.42
1.8	715	13.2	82	15.2	1.44	782	14.0	110	13.2	1.36
1.9	812	11.6	98	13.1	1.39	914	12.2	108	11.3	1.47
2.0	893	10.7	103	12.2	1.42	983	11.1	129	11.5	1.44
2.1	973	10.1	97	10.4	1.42	1054	10.3	134	11.1	1.45
2.2	1019	9.5	110	10.4	1.43	1149	9.7	123	10.5	1.44
2.3	1083	9.1	102	9.3	1.41	1214	9.2	134	9.3	1.46
2.4	1132	8.8	97	9.3	1.41	1285	8.7	137	8.7	1.46
2.5	1175	8.5	103	8.8	1.40	1339	8.4	140	8.5	1.47
2.6	1220	8.1	117	8.7	1.38	1388	8.0	149	8.2	1.47
2.7	1253	7.8	123	8.9	1.40	1431	7.8	154	7.9	1.44
2.8	1286	7.6	127	8.6	1.41	1481	7.6	149	7.8	1.43
2.9	1329	7.4	131	8.0	1.40	1498	7.4	163	7.8	1.43
3.0	1366	7.2	129	7.8	1.42	1538	7.3	159	7.7	1.42
3.1	1404	7.1	124	7.6	1.40	1578	7.1	158	7.6	1.43
3.2	1420	6.9	133	7.3	1.41	1633	7.0	148	7.4	1.42
3.3	1446	6.8	136	7.4	1.39	1662	6.8	159	7.3	1.43
3.4	1472	6.7	139	7.1	1.38	1693	6.6	162	7.1	1.44
3.5	1501	6.6	138	6.9	1.40	1720	6.5	166	6.9	1.44
3.6	1525	6.5	135	6.8	1.40	1749	6.5	158	6.8	1.44
3.7	1550	6.4	131	6.8	1.39	1771	6.4	168	6.6	1.44
3.8	1580	6.4	124	6.7	1.39	1789	6.3	173	6.5	1.43
3.9	1600	6.3	128	6.3	1.39	1808	6.2	179	6.6	1.43
4.0	1620	6.2	129	6.4	1.40	1831	6.1	187	6.5	1.43
4.1	1636	6.1	135	6.2	1.40	1853	6.0	190	6.4	1.42
4.2	1656	6.0	139	6.2	1.40	1865	6.0	204	6.1	1.43
4.3	1672	5.9	138	6.4	1.41	1887	5.9	209	6.0	1.43
4.4	1690	5.9	139	6.4	1.41	1907	5.8	210	5.9	1.43
4.5	1707	5.8	143	6.3	1.41	1926	5.7	217	5.8	1.43
4.6	1726	5.8	146	6.1	1.41	1948	5.7	214	5.8	1.43
4.7	1741	5.7	143	6.1	1.41	1963	5.6	221	5.7	1.43
4.8	1756	5.7	143	6.0	1.41	1982	5.6	227	5.6	1.42
4.9	1772	5.6	143	6.1	1.40	2010	5.5	232	5.4	1.42
5.0	1776	5.6	148	6.0	1.40	2029	5.5	223	5.4	1.42
5.1	1790	5.6	148	5.9	1.40	2048	5.4	220	5.5	1.42
5.2	1801	5.5	149	5.8	1.39	2066	5.4	219	5.4	1.41
5.3	1811	5.5	152	5.8	1.40	2089	5.3	215	5.4	1.41
5.4	1824	5.5	147	5.7	1.40	2098	5.3	223	5.3	1.41
5.5	1837	5.4	148	5.7	1.40	2109	5.3	223	5.3	1.41
5.6	1846	5.4	155	5.7	1.40	2131	5.2	225	5.3	1.40
5.7	1864	5.4	155	5.6	1.40	2152	5.1	232	5.2	1.40

5.8	1872	5.3	157	5.6	1.40	2170	5.1	230	5.1	1.40
5.9	1880	5.3	159	5.5	1.40	2179	5.1	242	5.1	1.40
6.0	1894	5.3	159	5.4	1.40	2193	5.0	240	5.1	1.40

* indicates the p-value (Poisson test) of the fold significantly less than 10^{-16}.
